# Comparative pharmacokinetics of valproic acid among Pakistani and South Korean patients: A population pharmacokinetic study

**DOI:** 10.1371/journal.pone.0272622

**Published:** 2022-08-24

**Authors:** Muhammad Usman, Qurrat-ul-Ain Shaukat, Muhammad Imran Khokhar, Rabiea Bilal, Rizwan Rasul Khan, Hafiz Asad Saeed, Mohsin Ali, Humaira Majeed Khan

**Affiliations:** 1 Institute of Pharmaceutical Sciences, University of Veterinary and Animal Sciences, Lahore, Pakistan; 2 Institute of Pharmacy, Faculty of Pharmaceutical and Allied Health Sciences, Lahore College for Women University, Lahore, Pakistan; 3 Ameer-ud-Din Medical College, Post-Graduate Medical Institute (PGMI), Lahore General Hospital, Lahore, Pakistan; 4 Gujranwala Medical College, Govt DHQ Hospital Gujranwala, Gujranwala, Pakistan; 5 CMH Lahore Medical College & IOD, NUMS, Lahore, Pakistan; 6 Department of Medicine, Aziz Fatima Medical & Dental College, Faisalabad, Pakistan; 7 Pak Emirates Military Hospital, Rawalpindi, Pakistan; 8 Department of Pharmacy Practice, Faculty of Pharmaceutical Sciences, Govt College University, Faisalabad, Pakistan; Bahauddin Zakariya University, PAKISTAN

## Abstract

**Purpose:**

The pharmacokinetics of valproic acid have been evaluated in a variety of populations however, the comparison in two different populations was yet to be reported. This study is aimed to compare the pharmacokinetics of valproic acid in Pakistani and South Korean patients.

**Method:**

The therapeutic drug monitoring (TDM) data of valproic acid from 92 Pakistani patients with 218 samples was combined with the data of 99 South Korean patients with 335 samples in order to form a pooled dataset of 191 patients with 553 samples. Population pharmacokinetic model was developed on NONMEM® software by using first order conditional estimation method for estimation of pharmacokinetic parameters. The influence of different covariates including ethnicity was evaluated the stepwise covariate modelling. The final model was evaluated for predictive performance and robustness by using goodness of fit plots and bootstrap analysis respectively.

**Results:**

The data was better described by one compartment model with first order elimination. The value for clearance (CL) of valproic in pooled data was 0.931 L/h with 43.4% interindividual variability (IIV) while volume of distribution (V_d_) was 16.6 L with 22.3% IIV. In covariate analysis, ethnicity and body weight were significant covariates for CL while body weight was also significant for V_d_.

**Conclusion:**

A significant difference in CL of valproic acid among Pakistani and South Korean patients was observed. The model can be used for the dose tailoring of valproic acid based on ethnicity and body weight of Pakistani and South Korean patients.

## Introduction

Valproic acid belongs to the class of broad spectrum anti-seizure drugs and is used for the treatment of generalized as well as partial seizures [1–4]. It has been proposed that valproic acid potentiates the effect of neurotransmitter gamma aminobutyric acid (GABA) in central nervous system (CNS) [1] however, the exact mechanism is yet to be explored [5]. Valproic acid is formulated in multiple dosage forms including oral tablets (simple tablet, sustained release and enteric coated), oral solutions, capsules, as well as intravenous injections [2]. For orally administered formulations, the time to achieve maximum plasma concentration (t_max_) is 10–12 h, 3–6 h and 1–2 h respectively for sustained release tablets, enteric coated tablets and oral solutions [1]. Valproic acid, being a weak acidic drug has high tendency (approximately 90% to 95%) to bind with albumin which becomes saturated at plasma concentration of 50 mg/L leading to disproportionate rise in plasma concentration [2]. Valproic acid exhibits high variability in volume of distribution (V_d_) ranging from 0.1 to 0.5 L/kg [2, 6] due to interindividual variability in protein binding based on age, gender, renal and hepatic status of the patient, co-morbidity, pregnancy and concomitantly administered drugs [1, 7]. Liver is the major rout of elimination for valproic acid and it is metabolized by glucuronidation, β-oxidation and metabolism through cytochrome P450 isoenzymes including CYP2C9, CYP2A6 and CYP2B6 [8, 9]. It also inhibits CYP2C9 isoenzyme which may lead to increased plasma concentration of co-administered drugs which are substrate for this enzyme [10, 11].

Valproic acid, being a narrow therapeutic index drug, requires a vigilant therapeutic monitoring in order to achieve safe and effective outcomes. The recommended range for target plasma concentration of valproic acid is 50 mg/L to 100 mg/L [5], however the upper level up to 125 mg/L is also recommended for the treatment of bipolar depression [12]. The plasma concentration of valproic acid below the recommended level may lead to therapeutic failure and the concentration above the upper limit can cause gastrointestinal side effects while serious side effects such as thrombocytopenia and tremors occur at concentration above 175–200 mg/L [13]. Since valproic acid exhibit high inter-individual variability owing to its elimination through hepatic rout, dose individualization can be achieved by population pharmacokinetic modeling [5]. A number of population pharmacokinetic studies have been performed to identify the effect of different covariates as well as inter-individual variability on pharmacokinetics of valproic acid [14–18] The effect of age has been specifically investigated on pharmacokinetics of valproic acid through population pharmacokinetic modelling approach [19, 20]. Pharmacokinetics of valproic acid have also been evaluated in population with different ethnicity such as China [21, 22], Serbia [23] and South Korea [24]. Since the major rout for elimination of valproic is hepatic, the genetic polymorphism may influence the elimination and ultimately the dose requirement in different patients with different ethnic origins. However, no study has been conducted to compare the population pharmacokinetics of valproic in different populations. This study is aimed to compare the pharmacokinetics of valproic acid in Pakistani and South Korean patients through population pharmacokinetic modelling by using the pooled dataset. In addition, this study was also aimed to identify the covariates responsible for inter-individual variability in pharmacokinetics of valproic acid in both populations.

## Materials and methods

### Study design and data collection

This was a multicenter, non-interventional comparative study performed by collecting the therapeutic drug monitoring (TDM) data of valproic acid from Pakistani patients and its comparison with data of already published population pharmacokinetic (popPk) model in South Korean patients [24]. The retrospective TDM data of 92 patients with 218 samples was received from Aziz Fatima Hospital Faisalabad, Pakistan. The ethical approval was sought from the institutional ethical committee of Aziz Fatima Medical & Dental College, Faisalabad, Pakistan vide letter No. DME/715-19. As the data was collected retrospectively and the samples were collected as their routine TDM process, there was no need to get the informed consent from the patients or their close relative. Serum concentrations of valproic acid were measured by immunoassay, Enzyme Linked Immunosorbent Assay (ELISA). The concentrations were measured at peak and trough concentration levels after the administration of standard dose which was decided by the attending physicians. The daily dose of valproic acid ranged from 500 mg to 1600 mg. The data of only those patients was included who received valproic acid intravenously at the dose decided by attending physician. In addition to serum concentrations the age, gender, body weight, height and body mass index (BMI) were also recorded. The data of 99 Korean patients was received from the corresponding author of already published article [24]. The TDM data of 92 patients with 218 samples was combined with the data of Korean patients in order to form a pooled dataset of 191 patients with 553 samples. The exploratory analysis of data was performed before the start of PK modeling process to record the demographics of patients in both populations.

### Population pharmacokinetic analysis

The actual process of popPK modelling was started by using Non-Linear Mixed Effect Modelling NONMEM® software version 7.4.4 (provided by Icon clinical research LLC New York, USA) with the aid of PsN toolkit [25]. The model management, execution, validation and reports generations was performed with the help of Pirana [26]. Initially, a base model was developed without introduction of any covariate. The data was analyzed by using a one-compartment as well as a two-compartment model and the suitable model was selected based on the objective function value (OFV) of model and visual inspection of goodness-of-fit plots of the base model. The inter-individual variability was evaluated by exponential model while the sampling and analysis errors were evaluated by additive error, proportional error and combined error models.

### Covariate analysis

After development of a base model, the influence of different covariates was observed on the pharmacokinetic parameters of valproic acid. This process was performed by stepwise covariate modelling (scm) in which the available covariates such as age, body weight, height, body surface area (BSA) and body mass index (BMI) as continuous covariates while gender and center (Pakistan & South Korea) as categorical covariates were included in the base model one by one and the drop in Objective Function Value (OFV) was observed with 0.05 level of significance which means that the drop in OFV by 3.84 points after the inclusion of a particular factors was considered significant for inclusion of that covariate in the model. The process was repeated again and again until no more factor was available for inclusion in full model. Then backward elimination process was started in which all the added factors in full model were removed one by one with a stricter criterion of level of significance (0.01) and the influence of that factor was considered significant for which the removal from full model resulted in increase in OFV by 6.63 points. This process was also repeated again and again until no more added factor remained available for elimination from full model and the obtained model was considered as final model.

### Comparison of Pharmacokinetic parameters

The pharmacokinetics parameters of valproic acid in Pakistani patients and South Korean patients were compared by using center (CENT) as a categorical covariate. The PK parameters of valproic acid are considered comparable if this covariate is proved non-significant during stepwise covariate modeling process. The values for V_d_ and CL of vancomycin were also compared in Pakistani and Korean patients through box and whisker plots.

### Model evaluation

The model obtained after the inclusion of all the significant covariates was considered as final model. The predictive performance of final model was evaluated by goodness-of-fit in which visual inspection of scatterplots of observed concentration (DV) vs. population predictions (PRED), DV vs. individual predictions (IPRED), Conditional Weighted Residuals (CWRES) vs. PRED and CWRES vs. Time after the dose was done. The robustness and stability of final model was assessed by using bootstrap analysis using PsN [25] in which final model was run for 1000 times. The datasets for bootstrap analysis were auto-generated in PsN by using different combination of the patients through repeated sampling. The pharmacokinetic parameter estimates of final model were compared with the median values of bootstrap results along with 95% confidence interval based on 2.5^th^ and 97.5^th^ percentile of distribution. The closeness of the final estimates with the bootstrap estimates were considered as the indication of final model stability.

## Results

### Patients and sampling data

The population pharmacokinetic (popPK) model of valproic acid was developed by using the pooled dataset comprising of 553 samples from 191 patients. Out of these 553 samples 218 (39.4%) samples were obtained from 92 Pakistani patients who were administered with valproic acid intravenously at Aziz Fatima Hospital Faisalabad. While the data of 335 samples (60.6%) was obtained from 99 South Korean patients [24]. The patients’ demographics and sampling data record is provided in Table 1. In pooled dataset of 191 patients, 127 (66.5%) were male patients and 64 (33.5%) were female patients. The median age of population in pooled dataset was 48 years with a range of 18 to 90 years while median body weight was 67 kg ranging from 40 to 101 kg. The height and BMI of patients ranged from 144 cm to 190 cm with median 167 cm and 15.6 to 37.6 kg/m^2^ with median of 23.2 kg/m^2^ respectively (Table 1).

**Table 1:** Demographics and sampling data of patients.

Patients and sampling data	Pakistani data	South Korean data	Pooled data
No. of Patients	92	99	191
Male (%)	64 (69.6%)	63 (63.6%)	127 (66.5%)
Female (%)	28 (30.4%)	36 (36.4%)	64 (33.5%)
Age (years)	54 (19–90)	44 (18–81)	48 (18–90)
Weight (kg)	71 (47–101)	60 (40–91)	67 (40–101)
Height (cm)	170 (148–190)	165 (144–187)	167 (144–190)
BMI (Kg/m^2^)0.	24.8 (20.1–37.6)	21.5 (15.6–31.6)	23.2 (15.6–37.6)
**Sample Data**			
No. of Samples	218	335	553
Single dose (mg)	1000 (500–1800)	1000 (500–1500)	1000 (500–1800)
Concentration (mg/L)	4.78 to 97.1	3.38 to 106.4	3.38 to 106.4

**Note:** The values are given in Median (Range) unless otherwise specified.

### Population PK modelling

The base model was developed by using both one compartment model and two compartment model with first order elimination. The values for OFV and the visual observation of goodness of fit plots revealed that the data is best described by one compartments model with first order elimination (SUBROUTIN ADVAN1 TRANS2). The estimation method used for calculation of PK parameters of valproic acid was first order conditional estimation with interaction (FOCE-I) while interindividual variability (IIV) was described by exponential random effect. The residual variability between predicted and observed concentration of valproic acid for estimation of sampling error and analysis error was described by proportional error model.

### Covariate analysis

During stepwise covariate modelling, the ethnicity (CENT) and body weight were proved as significant covariates for CL and reduced the OFV by 50.4 points after inclusion in the base model. The influence of body weight was also significant on Vd of valproic acid and resulted in further decrease in OFV by 32.8 points **(S1 Table)**. The values for CL of valproic acid and V_d_ in final model were 0.931 L/h and 16.6 L respectively. The Eqs 1 and 2 describe the influence of ethnicity on CL (CLCENT) of valproic acid in the final model.

$$\text{IF}\left(\text{CENT}.\text{EQ}.0\right)\;\text{CL}=0.931;\;\text{Most}\;\text{common}$$ (1)

$$\text{IF}\left(\text{CENT}.\text{EQ}.1\right)\;\text{CL}=\left(0.931+0.386\right)=1.317$$ (2)

Where; CENT.EQ.0 is for South Korean patients and CENT.EQ.1 is for Pakistani patients

The influence of body weight on CL can be described by Eq 3 as;

$${\text{CL}}_{\text{n}}=0.931\times {\left(1+0.0143\times \left({\text{WT}}_{\text{n}}–67\right)\right)}^{\eta 1}$$ (3)

Where; CL_n_ is clearance for n^th^ individual, η1 is the IIV for valproic acid CL and 0.931 is the median value of valproic acid CL for this pooled population. WT_n_ is the body weight of n^th^ individual and 67 is median body weight of pooled population.

The influence of body weight and age of the patients on V_d_ of valproic acid is quantified in Eq 4.

$${\text{V}}_{n}=16.6\times {\left(1+0.009\times \left({\text{WT}}_{\text{n}}–67\right)\right)}^{\eta 1}$$ (4)

Where; V_n_ is V_d_ of valproic acid for n^th^ individual with body weight WT_n_ in kg and age of AGE_n_ in years. While 16.6 and 67 are the median values of V_d_ and body weight of pooled population in liters and kg respectively.

### Comparison of Pharmacokinetic parameters

**Fig 1:**
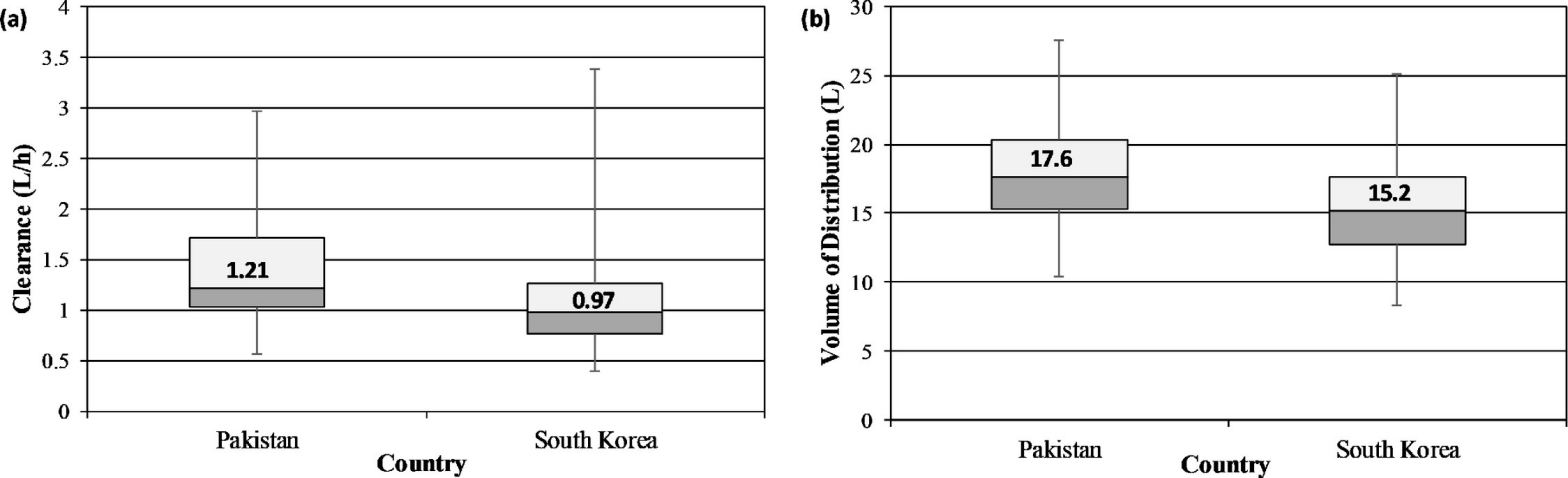
Box-plots showing CL (a) and V_d_ (b) of valproic acid among Pakistani and South Korean patients.

Ethnicity (CENT) was proved as a significant covariate for CL of valproic in Pakistani and South Korean patients which reveals that there is significant difference in CL of valproic in both populations. The median value for CL along with range in Pakistani and South Korean patients was 1.21 (0.55–2.96) L/h and 0.97 (0.39–3.38) L/h respectively while the median value of V_d_ along with range was 17.6 (10.4–27.5) L in Pakistani patients and 15.2 (8.36–25.1) L in South Korean patients. The comparison of CL and V_d_ of valproic acid in Pakistani and South Korean patients is given in (Fig 1A and 1B).

### Model evaluation

**Fig 2:**
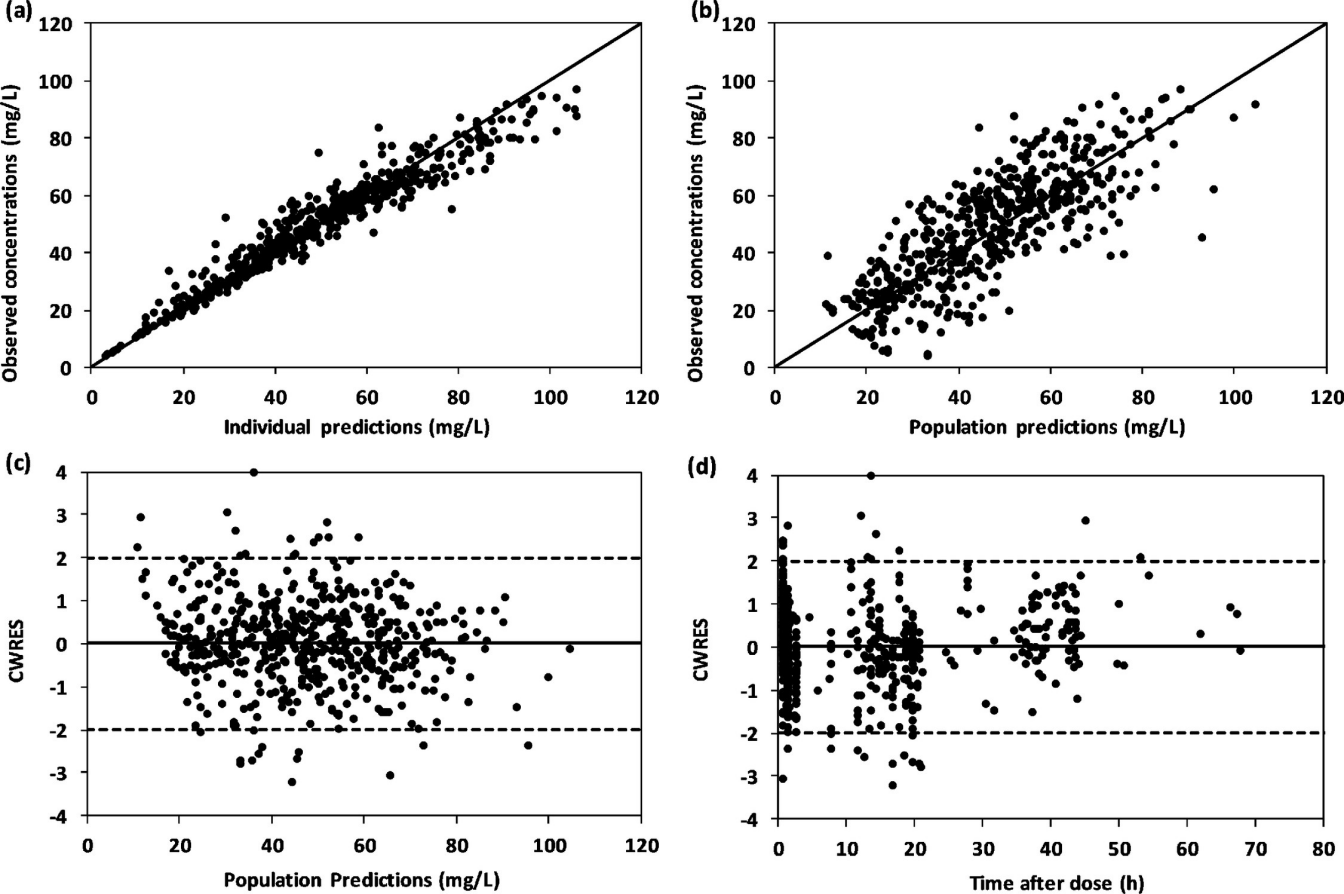
Goodness-of-fit plots of final model showing observed concentrations vs. individual predictions (a), observed concentrations vs. population predictions (b), CWRES vs. population predictions (c) and CWRES vs. time after dose (d).

The combined goodness of fit plots to evaluate the predictive performance of final model are shown in Fig 2. A uniform distribution of observed concentrations versus individual predictions as well as population prediction around the line of identity was observed in Fig 2A and 2B. The conditional weighted residuals (CWRES) were randomly distributed around zero line in scatterplots of CWRES versus population predictions as well as time after dose and more than 95% values were between the acceptable range of -2 to 2 as shown in Fig 2C and 2D). The stability of final model was confirmed by bootstrap estimates compared with the parameter estimates of final model. A close agreement was observed between parameter estimates of final model when compared with median values of 1000 bootstrap estimates along with 95% confidence interval (Table 2).

**Table 2:** Parameter estimates of final model compared with bootstrap estimates.

Parameter	Final estimate	RSE%	Bootstrap estimate	95% CI^a^
OFV	3344.6	-	3331.1	3156.5–3499.1
CL (L/h)^b^	0.931	5	0.932	0.844–1.024
V_d_ (L)^c^	16.6	2	16.6	15.9–17.3
Proportional Error	0.148	7	0.147	0.128–0.167
CL-CENT^d^	0.386	28	0.382	0.295–0.456
CL-WT^e^	0.0143	17	0.014	0.0094–0.019
V_d_-WT^f^	0.009	19	0.009	0.0056–0.0123
IIV CL (%)	43.4	13	42.7/	37.4–48.6
IIV V_d_ (%)	22.3	16	22.1	18.5–25.5

^a^ 95% Confidence interval of 1000 bootstrap estimates.

^b^ Clearance of valproic acid in South Korean patients and body weight of 67 kg.

^c^ Volume of distribution of valproic in patient with body weight 67 kg.

^d^ Difference of CL between South Korean and Pakistani patients.

^e^ Proportional change in CL with body weight.

^f^ Proportional change in V_d_ with body weight.

## Discussion

The identification of patients’ characteristics and magnitude of variability among the individuals that influence the pharmacokinetics of a drug in a particular population can be helpful in dose individualization. Valproic acid belongs to narrow therapeutic index drugs and vigilant dosing strategy is essential for individual patients in order to ensure the safe and effective outcome of therapy. Population pharmacokinetic modelling with analysis of covariates affecting the pharmacokinetics help in identification of the factors which should be considered for dose individualization. A number of population pharmacokinetic studies have been reported and about 85% studies were performed by using TDM data [5]. The development of population pharmacokinetic model by using sparse data is a unique feature of NONMEM offering a valuable advantage for pharmacokinetic analysis because the patients from whom sample collection is difficult are also the ones for whom the appropriate dose selection is critical [27].

The major rout for elimination of valproic acid is through hepatic biotransformation [8], therefore, the genetic polymorphism in its metabolizing enzymes CYP2C9 and CYP2C19 can also affect its CL and this has been observed in two studies [28, 29] however, only one study reported the significant influence of these isoenzymes on valproic acid CL [28]. The other covariates tested on pharmacokinetics of valproic acid in previous studies include age, gender, body weight, co-administered medication and dose of valproic acid [5]. Since the ethnic origin of the patients is a major factor for genetic polymorphism, therefore there was a need of evaluating the ethnicity on the pharmacokinetics of valproic acid. To the best of our knowledge, based on literature search, this is the first study of its kind to find the possible effect of ethnicity on valproic acid CL through population pharmacokinetic modelling in Pakistani and South Korean patients by using a pooled dataset.

The data was best described by compartment model which is in agreement with already reported models of valproic acid with only few exceptions [14, 30, 31] where the data was described by two compartment model. The estimated CL of valproic acid in our study was 0.931 L/h with IIV of 43.4% while the value of CL reported in previous studies is 0.206 L/h to 1.154 L/h with IIV ranging from 13.4% to 35.9% [5]. Therefore, the CL of valproic acid in our pooled dataset is similar to already values of CL however, IIV in our study is higher than previously reported studies may be due to a variety of patients from two populations. The value for V_d_ in our study was 16.6 L with IIV of 22.3% which is also in close agreement with previously reported studies where value of V_d_ ranged from 8.4 L to 23.3 L with IIV of 2.1% to 49% [5].

In covariate analysis, ethnicity and body weight were the significant covariate for CL of valproic acid. The influence of body weight on CL has also been reported in majority of studies [17, 21, 30, 32–34] however, no such study has been found which compares the pharmacokinetics of valproic acid in different ethnic groups through population pharmacokinetic modeling approach. The reason for variability in CL of valproic acid among Pakistani and Korean patients might be the fact that valproic acid is primarily eliminated from liver and there is high interindividual variability in metabolism based on the ethnicity. The V_d_ of valproic acid was also significantly influenced by body weight of the patients. The effect of weight on CL and V_d_ has also been reported in the Korean study the data of which has been added in this model for comparison [24]. Body weight and age have also been reported collectively in Serbian population [23]. The ethnicity was proved as significant covariate which reveals that the pharmacokinetics of valproic is significantly different among Pakistani and South Korean patients and the median value of CL in Pakistani patients (1.21 L/h) was higher than South Korean patients (0.97 L/h). The value for V_d_ was almost similar in both populations that is 17.6 L and 15.2 L in Pakistani and Korean patients may be due to comparable values of body weight and age of the populations which are the significant covariates.

The limitations in our study is that the less number of covariates have been tested because only these covariates were available in the data of South Korean population received from Prof. Dong-Seok Yim.

## Conclusion

A population pharmacokinetic model of valproic acid was developed by using the pooled dataset of Pakistani and South Korean patients with the aim to identify the possible difference in pharmacokinetic parameters of valproic acid in these two ethnicities and to devise tailored dosing. The clearance of valproic acid was influenced by body weight and ethnicity of the patients while volume of distribution was influenced by body weight of the patient. A significant difference in CL of valproic acid among Pakistani and South Korean patients was observed. The model can be used for the dose tailoring of valproic acid based on ethnicity and body weight of Pakistani and South Korean patients.

## Supporting information

S1 Table Results of stepwise covariate modeling. (TXT)

S1 Graphical abstract (TIF)
