# Supplementary figures and images for: Comparative pharmacokinetics of valproic acid among Pakistani and South Korean patients: A population pharmacokinetic study
Source: PLoS One. 2022 Aug 24;17(8):e0272622. doi: 10.1371/journal.pone.0272622 (PMC9401156; doi:10.1371/journal.pone.0272622)

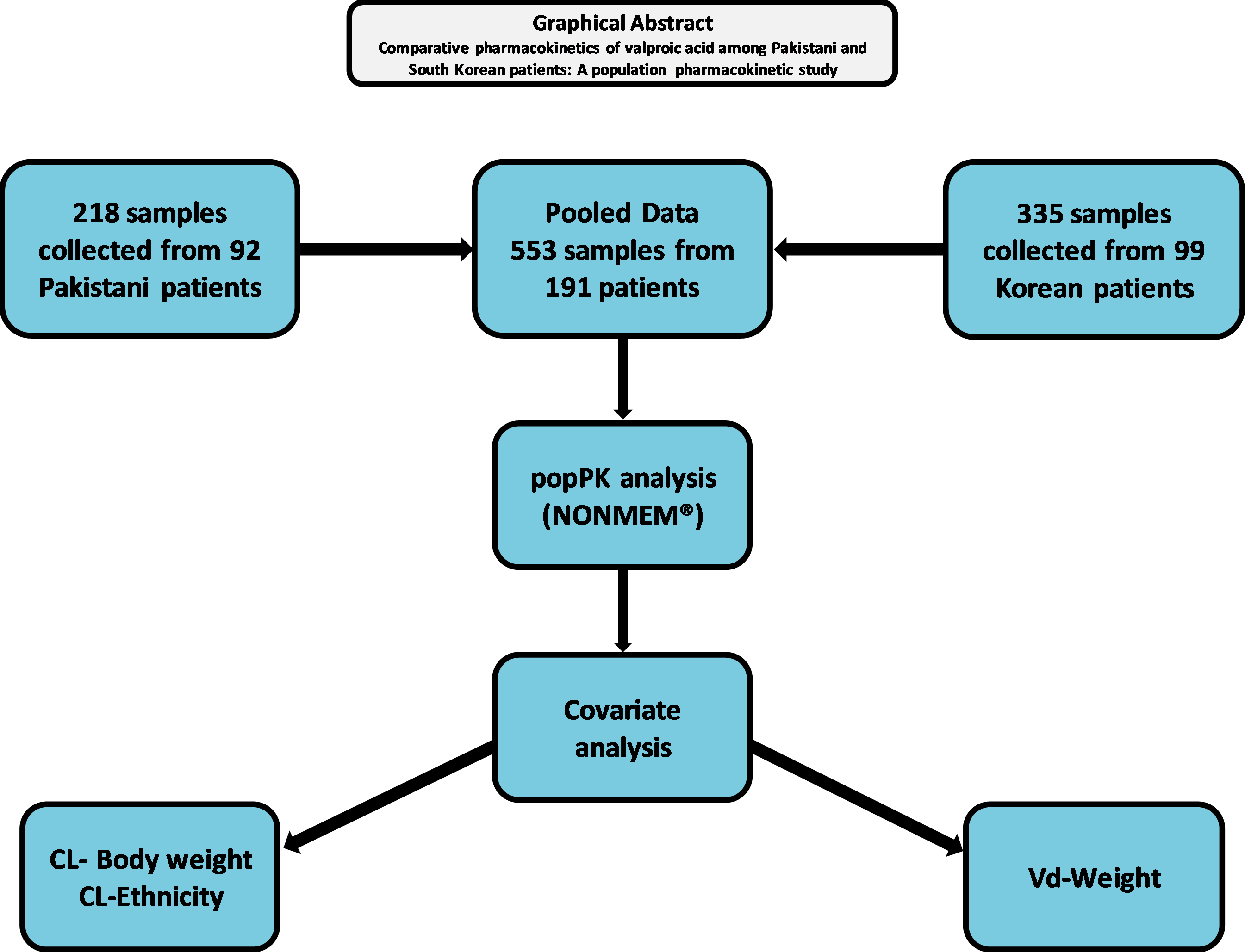

Supplement: S1 Graphical abstract — (TIF) [file pone.0272622.s002.tif]
